# Supplementary material for: The proteasome biogenesis regulator Rpn4 cooperates with the unfolded protein response to promote ER stress resistance
Source: eLife. 2019 Mar 13;8:e43244. doi: 10.7554/eLife.43244 (PMC6415940; doi:10.7554/eLife.43244)
Supplement: Supplementary file 1. [file elife-43244-supp1.docx]

**Supplementary Table 1.** Plasmids used in this study. GEM = Gal4DBD-EstR-Msn2TAD.

| Plasmid | Alias | Source | |
| --- | --- | --- | --- |
| pRS416-P_GAL1_ | pSS031 | Mumberg et al., 1994 | |
| pRS306-P_ADH1_-GEM | pDEP001 | Pincus et al., 2014 | |
| pNH605-P_ADH1_-GEM | pDEP151 | David Pincus | |
| pRS306-P_ADH1_-GEM-P_GAL1_ | pSS476 | this study | |
| pNH605-P_ADH1_-GEM-P_GAL1_ | pSS474 | this study | |
| pRS315-P_CPY_-ngCPY*-HA | pPW2181 | Peter Walter | |
| pRS315-P_GAL1_-CPY*-HA | pES67 | Spear and Ng, 2003 | |
| pRS306-P_ADH1_-GEM-P_GAL1_-ngCPY*-HA | pSS577 | this study | |
| pRS306-P_ADH1_-GEM-P_GAL1_-CPY*-HA | pSS953 | this study | |
| pRS305-HAC1-splicing-reporter | pDEP005 | Pincus et al., 2010 | |
| pRS305-P_ADH1_-Rtn1-FLAG-sfGFP | pSS421 | Szoradi et al., 2018 | |
| pNH605-P_ADH1_-GEM- P_GAL1_-FLAG-sfGFP | pSS525 | this study | |
| pNH605-P_ADH1_-GEM-P_GAL1_-ngCPY*-HA-sfGFP | pSS616 | this study | |
| pRS305-P_CPY_-CPY*(N479Q)-HA | pSS104 | Peter Walter | |
| pNH605-P_ADH1_-GEM-P_GAL1_-CPY*(N479Q)-HA-sfGFP | pSS562 | this study | |
| YEp13 | pSS471 | Michael Knop | |
| YEp13-RPN4 | pSS811 | this study | |
| YEp13-PDR1 | pSS813 | this study | |
| YEp13-SSZ1 | pSS812 | this study | |
| YEp13-MUM2 | pSS822 | this study | |
| YEp13-YAP1 | pSS814 | this study | |
| YEp13-CAD1 | pSS815 | this study | |
| YEp13-SAF1 | pSS744 | this study | |
| YEp13-NOP56 | pSS818 | this study | |
| YEp13-CDC48 | pSS816 | this study | |
| pRS306-P_ADH1_-GEM-P_GAL1_-∆ss-ngCPY*-HA | pSS678 | this study | |
| pRS306N-P_TEF1_-NES-Luciferase(DM)-mCherry | pSS637 | Szoradi et al., 2018 | |
| pRS303K-P_GPD_-TagBFP | pMAM227 | Michael Knop | |
| pRS303H-P_GPD_-TagBFP | pMAM245 | Michael Knop | |
| pRS304 | pSS003 | Sikorski and Hieter, 1989 | |
| pRS304-HAC1-splicing-reporter | pSS1023 | this study | |
| pNH604-4xP_HSE_-YFP | pDP122 | Zheng et al., 2016 | |
| pNH605-P_HSP12_-GFP | pDP012 | Pincus et al., 2014 | |
| pNH604-P_HSP12_-GFP | pSS1018 | this study | |
| pNH605-P_RPN4_-YFP | pSS975 | | Felix Boos |
| pNH605-P_RPN4_-(PDREm)-YFP | pSS977 | | Felix Boos |
| pNH605-P_RPN4_-(HSEm)-YFP | pSS976 | | Felix Boos |
| pFA6a-mNeonGreen-kanMX4 | pMAM375 | | Michael Knop |
| pNH605-P_RPN4_-mNeonGreen | pSS981 | | this study |
| pNH605-P_RPN4_-(PDREm)-mNeonGreen | pSS983 | | this study |
| pNH605-P_RPN4_-(YREm)-mNeonGreen | pSS987 | | this study |
| pNH605-P_RPN4_-(HSEm)-mNeonGreen | pSS982 | | this study |
| pNH605-P_RPN4_-(YREm,HSEm)-mNeonGreen | pSS984 | | this study |
