# Supplementary material for: The proteasome biogenesis regulator Rpn4 cooperates with the unfolded protein response to promote ER stress resistance
Source: eLife. 2019 Mar 13;8:e43244. doi: 10.7554/eLife.43244 (PMC6415940; doi:10.7554/eLife.43244)
Supplement: Supplementary file 2. [file elife-43244-supp2.docx]

**Supplementary Table 2.** Oligonucleotides used in this study.

| Oligonucleotide | Sequence |
| --- | --- |
| up_SacI_GAL | CAAGCATTTAGTAATGACTATCAAAgagctctagtacggattagaag |
| CYC_KpnI_down | ATAGCCTTATCAGATGATTCACCGGggtaccggccgcaaattaaag |
| EDY*_F2 | CTAGAACTAGTGGATCCCCCatgaaagcattcaccagtttac |
| EDY*_R | ATATCGAATTCCTGCAGCCCctaacccaaagaagcgtaatc |
| FLAG-sfGFP_F | CTAGAACTAGTGGATCCCCCgactacaaggacgacgatg |
| FLAG-sfGFP_R | ATATCGAATTCCTGCAGCCCctacttataaagctcgtcc |
| EDY*_tag_R | GTCATCGTCGTCCTTGTAGTCacccaaagaagcgtaatctg |
| FLAG for 1 | GACTACAAGGACGACGATGAC |
| FLAG-open_R | gggggatccactagtTCTAG |
| gap_RPN4_fw | CTATCGACTACGCGATCATGGCGACCACACCCGTCCTGTGtactgaattgtattttattctacc |
| gap_RPN4_rev | TGGCGCCGGTGATGCCGGCCACGATGCGTCCGGCGTAGAGggtttcataaaaaatccttaagg |
| gap_PDR1-2_fw | CTATCGACTACGCGATCATGGCGACCACACCCGTCCTGTGatgctaatctatgtacactacg |
| gap_PDR1-2_rev | TGGCGCCGGTGATGCCGGCCACGATGCGTCCGGCGTAGAGggcgcctttactggtggg |
| gap_SSZ1_fw | CTATCGACTACGCGATCATGGCGACCACACCCGTCCTGTGgctttgtaagcatggtttacc |
| gap_SSZ1_rev | TGGCGCCGGTGATGCCGGCCACGATGCGTCCGGCGTAGAGtgtacagactttatcgtatacg |
| gap_MUM2_fw | CTATCGACTACGCGATCATGGCGACCACACCCGTCCTGTGgtgtatgatcaaatctatcgag |
| gap_MUM2_rev | TGGCGCCGGTGATGCCGGCCACGATGCGTCCGGCGTAGAGtgccttgtatcatcatgaacg |
| gap_YAP1_fw | CTATCGACTACGCGATCATGGCGACCACACCCGTCCTGTGaggtaccatatacgaagatcg |
| gap_YAP1_rev | TGGCGCCGGTGATGCCGGCCACGATGCGTCCGGCGTAGAGaatcattattggtactattgcac |
| gap_CAD1_fw | CTATCGACTACGCGATCATGGCGACCACACCCGTCCTGTGagtgtttcttcaatagttaatcg |
| gap_CAD1_rev | TGGCGCCGGTGATGCCGGCCACGATGCGTCCGGCGTAGAGtgtaggtatccacttgtatcg |
| gap_SAF1_fw | CTATCGACTACGCGATCATGGCGACCACACCCGTCCTGTGtgcttaatatatacccaatttgc |
| gap_SAF1_rev | TGGCGCCGGTGATGCCGGCCACGATGCGTCCGGCGTAGAGaatattgagaagtgaagggag |
| gap_NOP56_fw | CTATCGACTACGCGATCATGGCGACCACACCCGTCCTGTGaatattaaagcagagtacattgc |
| gap_NOP56_rev | TGGCGCCGGTGATGCCGGCCACGATGCGTCCGGCGTAGAGtggcgatcacagaatgtcag |
| gap_CDC48_fw | CTATCGACTACGCGATCATGGCGACCACACCCGTCCTGTGttacactcacaccaatgatgg |
| gap_CDC48_fw | TGGCGCCGGTGATGCCGGCCACGATGCGTCCGGCGTAGAGtcaaatcgtgattttaaatgtcg |
| CPY deltaSS fw | CCATGgccatctcattgcaaagacc |
| CPY deltaSS rev | ATGAGATGGCcatgggggatccactagttc |
| knock-in URA3 fw | ATTTATGGTGAAGGATAAGTTTTGACCATCAAAGAAGGTTagcttgtctgtaagcggatg |
| knock-in URA3 rev | GAAGCTTTTTCTTTCCAATTTTTTTTTTTTCGTCATTATAcatgttctttcctgcgttatcc |
| Yep13 fw | CGTGTATGAAATCTAACAATGC |
| Yep13 rev | AAGGAATGGTGCATGCAAGG |
| pPCR_RPN4_fw | AGTGAAGCAACGGCCAACG |
| qPCR_RPN4_rev | GTCTTCTGCAATGGGGTTTCG |
| qPCR_TAF10_fw | GGATCAGGTCTTCCGTAGCG |
| qPCR_TAF10_rev | AGGCTGTTGCTGTCCTTGC |
| qPCR_KAR2_fw | ATTCCACCAGCACCAAGAGG |
| qPCR_KAR2_rev | ACCAGTTCCCTTATCTGTGGC |
| qPCR_SIL1_fw | AGAAGACATGAAAGCATCGCC |
| qPCR_SIL1_rev | CCTCCAATCTGGCAATGTCC |
| pRS304-Eco-Hac1-SR fw | TATCGATAAGCTTGATATCGtgcgctaccttcatagtcgg |
| pRS304-Eco-Hac1-SR rev II | TGGATCCCCCGGGCTGCAGGgttgaagtagcacacactaacc |
| Xho-neon | AAActcgagATGGTTTCTAAGGGTGAAGAAG |
| knock-in LEU2 fw | Aagatccatgtataatcttcattattacagccctcttgacagcttgtctgtaagcggatg |
| knock-in LEU2 rev | taccctatgaacatattccattttgtaatttcgtgtcgcatgttctttcctgcgttatcc |
| neon-BamHI | TTTggatccTTACTTGTACAATTCGTCCATAC |
| NheI-YRE fw | aaaGCTAGCatagccgtttacagtgatgg |
| NheI-YRE rev | aaaGCTAGCtagcgatcacgtgattatcc |
