# Supplementary material for: The proteasome biogenesis regulator Rpn4 cooperates with the unfolded protein response to promote ER stress resistance
Source: eLife. 2019 Mar 13;8:e43244. doi: 10.7554/eLife.43244 (PMC6415940; doi:10.7554/eLife.43244)
Supplement: Supplementary file 3. [file elife-43244-supp3.docx]

**Supplementary File 3.** Yeast strains used in this study. For strains with extra-chromosomal plasmids only the genotype of the parental strains without plasmid is given. GEM = Gal4DBD-EstR-Msn2TAD, SR = splicing reporter.

| Strain | Relevant genotype | Source |
| --- | --- | --- |
| SSY122 | *ADE2 leu2-3,112 trp1-1 ura3-1 his3-11,15 MAT a* | Szoradi et al., 2018 |
| SSY1338 | *URA3::P_ADH1_-GEM-P_GAL1_-ngCPY*-HA* | this study |
| SSY2491 | *URA3::P_ADH1_-GEM-P _GAL1_-ngCPY*-HA LEU2::HAC1-SR* | this study |
| SSY2489 | *URA3::P_ADH1_-GEM-P_GAL1_-CPY*-HA* | this study |
| SSY2490 | *URA3::P_ADH1_-GEM-P_GAL1_-CPY*-HA LEU2::HAC1-SR* | this study |
| SSY2364 | *Sec63-mCherry::TRP1 LEU2::P_ADH1_-GEM-P_GAL1_-ngCPY*-HA-sfGFP* | this study |
| SSY2363 | *Sec63-mCherry::TRP1 LEU2::P_ADH1_-GEM-P_GAL1_-* *CPY*(N479Q)-HA-sfGFP* | this study |
| SSY1337 | *URA3::P_ADH1_-GEM-P_GAL1_* | this study |
| SSY1340 | *hac1∆::HIS3 URA3::P_ADH1_-GEM-P_GAL1_* | this study |
| SSY1341 | *hac1∆::HIS3 URA3::P_ADH1_-GEM-P_GAL1_-ngCPY*-HA* | this study |
| SSY2748 | *hac1∆::HIS3 URA3::P_ADH1_-GEM-P_GAL1_-ngCPY*-HA rpn4::nat-P_CYC1_-RPN4* | this study |
| SSY1062 | *hac1∆::HIS3* | this study |
| SSY784 | *rpn4∆::HIS3* | this study |
| SSY2144 | *rpn4∆::HIS3 hac1∆::hph* | this study |
| SSY2145 | *rpn4∆::HIS3 URA3::P_ADH1_-GEM-P_GAL1_-ngCPY*-HA* | this study |
| SSY2745 | *rpn4∆::nat hac1∆::HIS3 URA3::P_ADH1_-GEM-P_GAL1_-ngCPY*-HA* | this study |
| SSY795 | *HIS3::P_GPD_-TagBFP-hph* | Szoradi et al., 2018 |
| SSY2357 | *HIS3::P_GPD_-TagBFP-hph LEU2::HAC1-SR* | this study |
| SSY853 | *rpn4∆::HIS3 HIS3::P_GPD_-TagBFP-hph* | this study |
| SSY2359 | *rpn4∆::HIS3 HIS3::P_GPD_-TagBFP-hph LEU2::HAC1-SR* | this study |
| SSY1545 | *RPN4-3HA::kan* | this study |
| SSY2147 | *hac1∆::hph RPN4-3HA::kan* | this study |
| SSY002 | *ade2-1 leu2-3,112 trp1-1 ura3-1 his3-11,15 MAT alpha* | Peter Walter |
| SSY1587 | *sec65-1, ura3-52, trp1-1,leu2-3,112, ade2, his3-11 MAT alpha* | Stirling et al., 1992 |
| SSY2452 | *ade2-1 LEU2::HAC1-SR* | this study |
| SSY2453 | *sec65-1 LEU2::HAC1-SR* | this study |
| SSY2444 | *URA3::P_ADH1_-GEM-P_GAL1_-∆ss-ngCPY*-HA* | this study |
| SSY1521 | *HIS3::P_GPD_-TagBFP-hph ura3::P_TEF1_-Luciferase(DM)-mCherry-nat* | Szoradi et al., 2018 |
| SSY2739 | *hac1∆::HIS3 URA3::P_ADH1_-GEM-P_GAL1_-ngCPY*-HA TRP1::4xP_HSE_-GFP* | this study |
| SSY2740 | *hac1∆::HIS3 URA3::P_ADH1_-GEM-P_GAL1_-ngCPY*-HA TRP1::P_HSP12_-GFP* | this study |
| SSY2741 | *hac1∆::HIS3 URA3::P_ADH1_-GEM-P_GAL1_-ngCPY*-HA leu2::HAC1-SR-TRP1* | this study |
| SSY1482 | *tpk1/2/3-as* | David Pincus |
| SSY1484 | *tpk1/2/3-as HIS3::P_GPD_-TagBFP-kan* | this study |
| SSY2621 | *tpk1/2/3-as HIS3::P_GPD_-TagBFP-kan LEU2::P_RPN4_-mNeonGreen* | this study |
| SSY2622 | *tpk1/2/3-as HIS3::P_GPD_-TagBFP-kan LEU2::P_RPN4_-(HSEm)-mNeonGreen* | this study |
| SSY2629 | *tpk1/2/3-as HIS3::P_GPD_-TagBFP-kan LEU2::P_RPN4_-(PDREm)-mNeonGreen* | this study |
| SSY2630 | *tpk1/2/3-as HIS3::P_GPD_-TagBFP-kan LEU2::P_RPN4_-(YREm)-mNeonGreen* | this study |
| SSY1483 | *tpk1/2/3-as msn2∆::nat msn4∆::TRP1* | David Pincus |
| SSY1485 | *tpk1/2/3-as msn2∆::nat msn4∆::TRP1 HIS3::P_GPD_-TagBFP-kan* | this study |
| SSY2623 | *tpk1/2/3-as msn2∆::nat msn4∆::TRP1 HIS3::P_GPD_-TagBFP-kan LEU2::P_RPN4_-mNeonGreen* | this study |
| SSY2624 | *tpk1/2/3-as msn2∆::nat msn4∆::TRP1 HIS3::P_GPD_-TagBFP-kan LEU2::P_RPN4_-(HSEm)-mNeonGreen* | this study |
| SSY2632 | *tpk1/2/3-as msn2∆::nat msn4∆::TRP1 HIS3::P_GPD_-TagBFP-kan LEU2::P_RPN4_-(PDREm)-mNeonGreen* | this study |
| SSY2633 | *tpk1/2/3-as msn2∆::nat msn4∆::TRP1 HIS3::P_GPD_-TagBFP-kan LEU2::P_RPN4_-(YREm)-mNeonGreen* | this study |
| SSY2634 | *tpk1/2/3-as msn2∆::nat msn4∆::TRP1 HIS3::P_GPD_-TagBFP-kan LEU2::P_RPN4_-(YREm,HSEm)-mNeonGreen* | this study |
